# Serum Neurofilament Light Chain and Glial Fibrillary Acidic Protein as Differential Biomarkers of Response to Dimethyl Fumarate and Ocrelizumab in Multiple Sclerosis

**DOI:** 10.3390/ijms27031441

**Published:** 2026-01-31

**Authors:** Alessandra Mingione, Andrea Corona, Corinne Monzani, Alen Zollo, Carola Cirocco, Tiziana Zaccone, Mariangela Scavone, Gian Marco Podda, Paola Signorelli, Monica Miozzo, Alberto Priori, Filippo Martinelli-Boneschi

**Affiliations:** 1 Laboratory of Precision Medicine of Neurological Diseases, Department of Health Science, University of Milan, 20142 Milan, Italy; 2 Aldo Ravelli Center for Neurotechnology and Experimental Brain Therapeutics, Department of Health Sciences, Università degli Studi di Milano, 20142 Milan, Italy; 3 Clinical Neurology Unit, ASST Santi Paolo e Carlo, 20142 Milan, Italy; 4 Laboratory of Haemostasis and Thrombosis, Dipartimento di Scienze della Salute, Università degli Studi di Milano, 20142 Milan, Italy; 5 Division of Medicina Generale SP, ASST Santi Paolo e Carlo, 20142 Milan, Italy; 6 Biochemistry Laboratory, IRCCS Policlinico San Donato, 20097 Milan, Italy; 7 Medical Genetics, Department of Health Sciences, Università degli Studi di Milano, 20142 Milan, Italy; 8 Medical Genetics Unit, ASST Santi Paolo e Carlo, 20142 Milan, Italy

**Keywords:** sNfL, sGFAP, SIMOA, multiple sclerosis, Dimethyl fumarate, Ocrelizumab

## Abstract

Multiple sclerosis (MS) is a chronic inflammatory and neurodegenerative disease. Quantifying neuronal damage is a critical step for patient care. Neurofilament light chain (sNfL) and glial fibrillary acidic protein (sGFAP) are the most promising serum biomarkers reflecting neuronal damage and astroglial activation, respectively. This study analyzed sNfL and sGFAP in 177 MS patients and 71 healthy controls (HCs) using SIMOA technology, classifying patients as responders (Rs) or non-responders (NRs) based on “No Evidence of Disease Activity 3” (NEDA-3) status during two years of treatment. Longitudinal analyses were performed for Dimethyl fumarate (DMF) and Ocrelizumab (OCRE) treatment. Biomarker–age correlation analysis in HCs confirmed correlation between both NfL and GFAP, with age and cut-off values specific for age decades being calculated. Both biomarkers were higher in MS patients compared to HCs. sNfL showed a significant increase in NR patients overall. In contrast, sGFAP was elevated in the low-to-moderate-efficacy treatment agents (LETAs) NR group and also in the DMF NR subgroup, suggesting that it monitors persistent astrogliosis. Longitudinal analysis showed that both biomarkers decreased during DMF treatment after one year. During OCRE treatment, sNfL rapidly reduced to HC levels within one year, while sGFAP decreased only after two years. This highlights that OCRE acts differently on the pathological processes linked to the two biomarkers.

## 1. Introduction

Multiple sclerosis (MS) is a chronic inflammatory and neurodegenerative disease. Accurately detecting and quantifying neuronal damage is an essential step for patient monitoring and treatment.

Neurofilaments (NFs) and glial fibrillary acidic protein (GFAP) are two key proteins of the CNS cytoskeleton that have emerged as promising biomarkers in MS.

Neurofilaments light chain (NfL) are neuron-specific intermediate filaments and their release into the cerebrospinal fluid (CSF) and blood reflects axonal damage [1]. Increased levels of NfL in the CSF and serum in patients with multiple sclerosis have been associated with active disease and disability progression [2,3,4].

Several studies have demonstrated a decrease in serum NfL (sNfL) levels following the initiation of disease-modifying therapies (DMTs), supporting its potential role as a biomarker for evaluating treatment effectiveness [2,5,6,7].

GFAP is an intermediate filament protein found predominantly in astrocytes. In MS, inflammation and axonal injury lead to astrogliosis, a process where astrocytes become reactive and proliferate [8]. This process results in an increase in GFAP expression. Increased GFAP levels have been related to predicting and assessing MS progression [9,10,11]. The impact of DMTs on serum GFAP (sGFAP) levels in patients remains to be established. sGFAP complements the sNfL, which is more indicative of acute inflammatory activity and relapses.

The combination of these biomarkers could be a significant advance toward personalized medicine in multiple sclerosis.

DMTs aim to control disease activity and reduce disease progression. The therapies are divided into two main categories based on their effectiveness: low–moderate effectiveness treatment agents (LETAs) and high-efficacy treatment agents (HETAs) [12]. Among the LETAs, one of the most used is Dimethyl fumarate (DMF), an immunomodulatory drug that activates the Nrf2 antioxidant pathway, increases regulatory T cells, and limits immune cell CNS migration [12]. Among the HETAs, one of the most effective is Ocrelizumab (OCRE). This agent is a humanized monoclonal antibody that works by selectively targeting and depleting B-cells that express CD20 protein on their surface, reducing the number of immune cells that can cause inflammation and damage to the central nervous system, thereby slowing disease progression and reducing the frequency of relapses [12,13,14]. However, there is a lack of measurable predictive factors in serum for clinical response to OCRE and DMF in MS patients. A variable number of patients show ongoing inflammatory activity or disease progression, indicating that there may be a suboptimal response to DMF or OCRE, resulting in a change in treatment. The efficacy of serum NfL and GFAP in monitoring response to treatment with Ocrelizumab and Dimethyl fumarate is currently unclear, and evidence in the literature is insufficient.

The use of sNfL and sGFAP in clinical practice is limited primarily by the lack of cut-off values for daily patient monitoring. Some studies have shown that NfL levels increase with age and BMI, making it difficult to establish optimal cut-off values [15,16,17]. Given the varying cut-off values reported in the literature, first we determined the sNfL and sGFAP levels in our cohort of control subjects [15,16,17,18,19].

Our study aims to evaluate variations in sNfL and sGFAP levels in multiple sclerosis patients treated with Dimethyl fumarate or Ocrelizumab and to ascertain the value of both serum biomarkers in predicting response to treatment.

For this purpose, patients were classified as responders or non-responders to current therapy according to clinical and radiological response, measured by the “No Evidence of Disease Activity 3” (NEDA3) status (absence of clinical relapses, absence of gadolinium-enhancing or new/enlarging T2 lesions on brain and spinal cord MRI and absence of confirmed disability progression) during two years of treatment [20].

## 2. Results

### 2.1. NfL and GFAP–Age Correlation Analysis in Healthy Subjects

**Figure 1:**
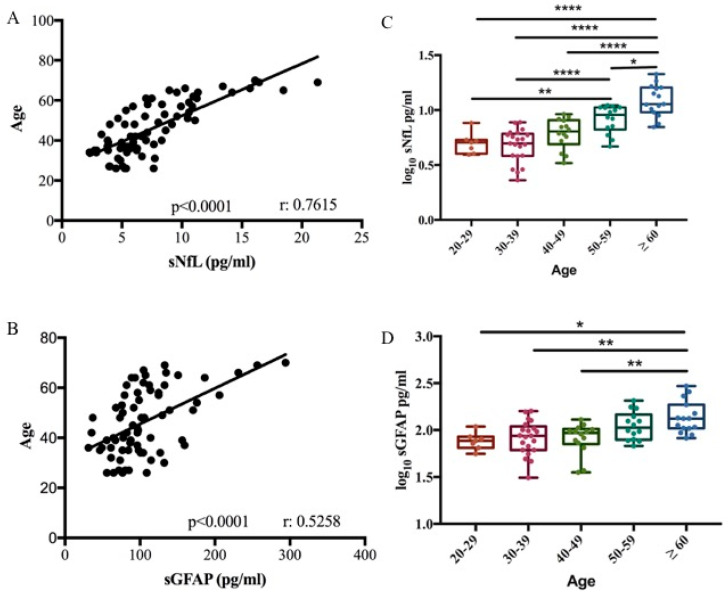
sNfL and sGFAP association with age. Correlation between sNfL (**A**) and sGFAP (**B**) levels with age in 71 healthy controls (Pearson correlation r = 0.7615, R^2^ = 0.58, *p* < 0.0001 for NfL) (Pearson correlation r = 0.5258, R^2^ = 0.28, *p* < 0.0001 for GFAP). Box plots of log_10_-transformed serum sNfL (**C**) and sGFAP (**D**) levels stratified according to age decade (one-way ANOVA, Bonferroni’s multiple comparisons test; * *p* = 0.035; ** *p* < 0.01; **** *p* < 0.0001).

We performed a biomarker–age correlation analysis in 71 healthy controls (HCs). A correlation between both sNfL and sGFAP with age was confirmed in our cohort (Pearson correlation r = 0.7615, *p* < 0.0001; r = 0.5258, *p* < 0.0001, respectively) (Figure 1A,B). Based on the relationship between the two biomarkers and age, the control group was divided into different age decades to define cut-off values. Biomarker levels were subjected to log_10_ transformation to normalize their distribution, as confirmed by the Shapiro–Wilk test (*p* > 0.05). Cut-off values for the healthy control cohort were then calculated as the mean + 2 standard deviations (SD) of the log-transformed data. Finally, these values were back-transformed to the original scale using the antilogarithm for clinical interpretation. No significant difference was observed in sNfL and sGFAP levels across the third (median 5.07 pg/mL, range 3.93–7.65 and median 76.82 pg/mL, range 55.8–109.04, respectively), fourth (median 4.97 pg/mL, range 2.29–7.74 and median 86.81 pg/mL, range 31.11–156.27, respectively), and fifth decades (median 6.39 pg/mL, range 3.29–9.17 and median 93.49 pg/mL, range 35.39–129.73, respectively) (Table 1 and Table 2). sNfL levels significantly increased in the sixth decade (9.08 pg/mL, range 4.67–11.08) compared to the third and fourth decade (*p* < 0.05 and *p* < 0.001). Finally, sNfL significantly increased in subjects ≥ 60 years old (median 11.34 pg/mL, range 7.01–21.31) compared to all other decades (*p* < 0.0001) (Figure 1C). GFAP levels increased significantly in subjects ≥ 60 years old (median 132.83 pg/mL, range 82.20–294.21) compared to third, fourth, and fifth decades (*p* < 0.01) (Figure 1D).

The thresholds of the measured values, related to each decade, were determined by calculating the mean plus two standard deviations (SDs), as presented in Table 1 and Table 2.

Based on the correlation data obtained, the results of the subsequent analyses were always corrected for age.

### 2.2. Measurement of sNfL and sGFAP in MS Patients and Healthy Controls

Serum levels of NfL and GFAP were measured in 177 patients and 71 healthy controls. Demographic variables and baseline data are detailed in Table 3. Over a median time period of 2 years, thirty-eight patients (22.5%) were non-responders to the ongoing therapy. Eighty-seven percent of the patients were on treatment at the time of sample collection. Specifically, 75 patients (43.3%) were receiving treatment with LETAs, while 98 patients (56.64%) were receiving treatment with HETAs. The median Expanded Disability Status Scale (EDSS) score was 2.5, with a range between 0 and 8.5. The median Multiple Sclerosis Severity Score (MSSS) was 3.86, with a range between 0.14 and 9.7.

**Figure 2:**
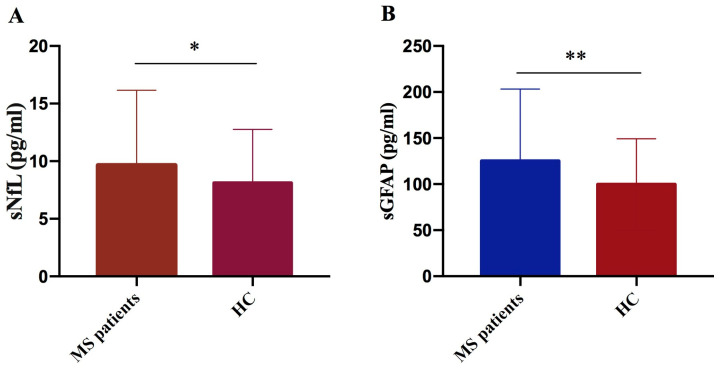
Comparison of NfL and GFAP concentrations in MS patients versus controls. NfL (**A**) and GFAP (**B**) levels were significantly higher in MS patients compared to controls (HC) (sNfL * *p* = 0.024; GFAP ** *p* = 0.004).

We performed a comparative analysis of biomarker serum levels, measured using SIMOA technology in a cohort of 177 MS patients and 71 healthy controls. We observed significant elevation in both sNfL and sGFAP levels in MS patients compared to controls (*p* = 0.024 and *p* = 0.004, respectively) (Figure 2A,B).

Linear regression analysis was performed in 177 MS patients to investigate the association between sNfL and sGFAP levels and disease severity. The disease disability indicator was calculated as the EDSS, and the disease severity indicator was calculated as Multiple Sclerosis Severity Score (MSSS); the analysis was adjusted for age. The results revealed a non-significant increasing trend of GFAP levels in relation to the degree of disability and disease severity (Table 4). While not statistically significant, the observed trends suggest that sGFAP levels may increase with greater disease burden. Instead, the positive correlation between sNfL, EDSS, and MSSS is very weak.

### 2.3. sNfL and sGFAP Levels in Treatment Responders and Non-Responders

Serum levels of NfL and GFAP were measured in 152 patients undergoing different therapies. Patients were categorized into two groups: responders or non-responders to the therapy at the time of sampling. Patients were defined as responders based on maintenance of “NEDA-3”: absence of clinical relapses, gadolinium-enhancing, or new/enlarging T2 lesions on brain and spinal MRI and absence of CDP (confirmed disability progression) in the first two years of treatment. The characteristics of the two groups are shown in Table 5.

**Figure 3:**
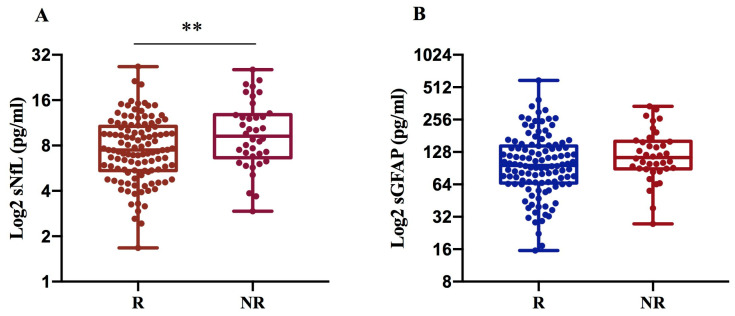
sNfL and sGFAP levels according to treatment response in 152 MS patients. Log2-transformed sNfL (**A**) and GFAP values (**B**) were compared between R and NR MS patients. NfL levels were significantly increased in non-responders to the current therapy (** *p* = 0.007) (**A**). R: responder; NR: non-responder.

We found that sNfL levels are significantly higher in NR (10.6 pg/mL) compared to R patients (8.4 pg/mL) with *p* = 0.007, while sGFAP levels exhibit only an increasing trend in NRs (134.6 pg/mL) compared to Rs (119 pg/mL), (Figure 3A,B).

To evaluate any differences in sNfL and sGFAP levels between different treatment strategies, we compared R and NR patients on low–moderate effectiveness treatment agents (LETAs) and those on high-efficacy treatment agents (HETAs), separately (see Table 6).

**Figure 4:**
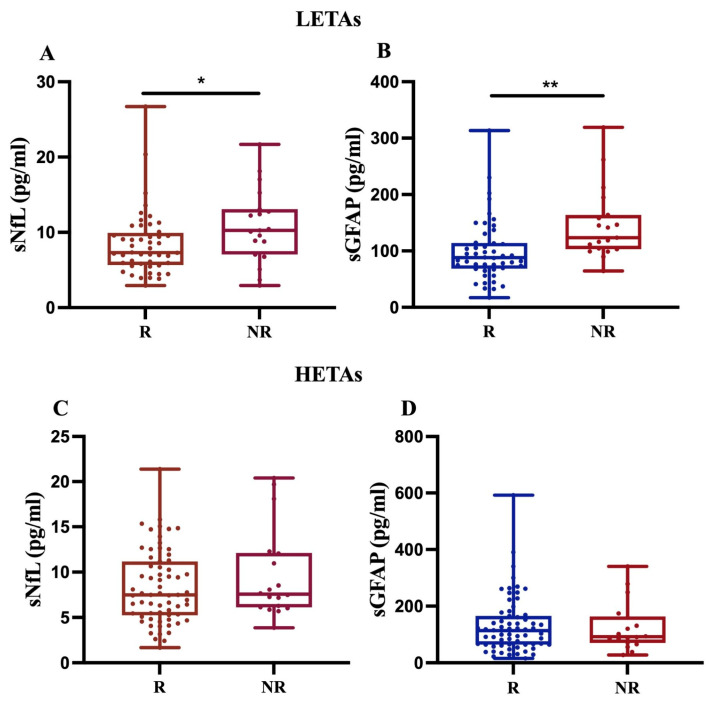
Differential sNfL and sGFAP levels in response to LETAs or HETAs treatment. sNfL and sGFAP levels were measured in 68 patients treated with LETAs (**A**,**B**) and 84 patients treated with HETAs (**C**,**D**). The results show a significant increase in both sNfL and sGFAP levels only in NR patients on LETAs (**A**,**B**) (* *p* = 0.024; ** *p* = 0.009).

First, our findings revealed that, in 68 MS patients treated with LETAs, both sNfL and sGFAP levels significantly increased in NR compared to R (*p* < 0.05 and *p* < 0.01, respectively) (Figure 4A,B). In contrast, for 84 MS patients on HETAs, the data showed an increasing trend only in sNfL levels in NR compared to R (Figure 4C,D). Given that we had few MS patients treated with rare drugs (methotrexate, *n* = 1; azathioprine, *n* = 2), we performed sensitivity analyses by excluding these patients, and the analyses did not change the statistical significance or direction of the main findings.

Moreover, as expected, patients on LETAs showed a lower median EDSS score of 2 (range 0–8.5) compared to patients on HETAs, who had a median EDSS of 3 (range 1–7.5). This difference was highly significant (*p* < 0.001).

Similarly, the median Multiple Sclerosis Severity Score (MSSS) was lower for LETA patients at 3 (0.14–9.77) compared to HETA patients, who showed a higher median MSSS of 5.24 (range 0.72–9.57). This disparity in severity was also strongly significant (*p* < 0.001).

### 2.4. NfL and GFAP Levels in Dimethyl Fumarate- and Ocrelizumab-Treated Patients

Based on the distinct data obtained from patients treated with LETAs and HETAs, we decided to measure NfL and GFAP levels in two specific subgroups. The first group of 24 patients was treated with Dimethyl fumarate (DMF), a drug with low–moderate efficacy, and the second group of 49 patients with Ocrelizumab (OCRE), a drug with high efficacy (Table 7).

**Figure 5:**
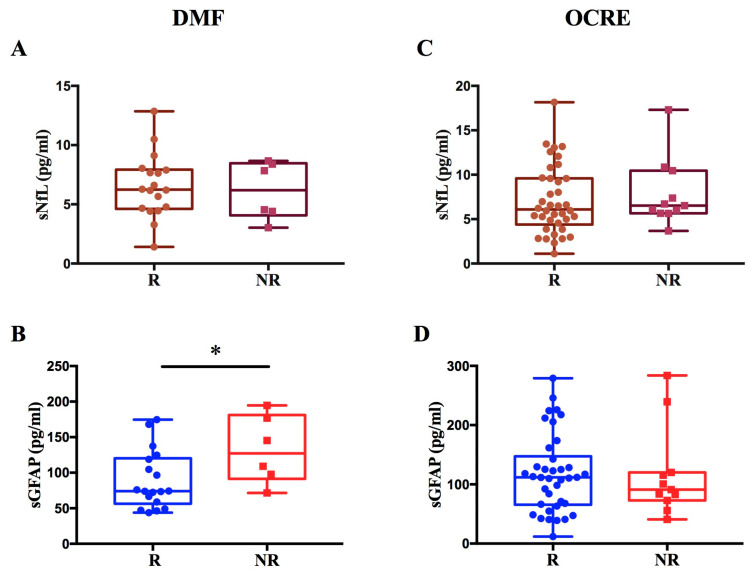
Evaluation of sNfL and sGFAP levels in response to DMF or OCRE treatment. The figure illustrates the levels of sNfL and sGFAP in MS patients treated with DMF or OCRE, classified as responders (R) or non-responders (NR). Panels (**A**,**B**) display sNfL and sGFAP values for patients treated with DMF, revealing a significant increase in GFAP levels in non-responder patients (* *p* = 0.036; β = 0.44) (**B**). Panels (**C**,**D**) show the sNfL and sGFAP values for patients treated with Ocrelizumab.

Regarding patients treated with DMF, we observed a significant increase in GFAP levels in non-responders compared to responders to therapy (*p* = 0.036; β = 0.44) (Figure 5A,B). We found no difference in sNfL levels between R and NR to DMF.

In contrast, for patients treated with OCRE, no significant difference was found in sNfL and sGFAP levels between non-responders and responders (Figure 5C,D).

These findings are consistent with the results from the entire patient cohort, which showed a modulation of sNfL and sGFAP levels exclusively among responders and non-responders on LETAs.

To assess the role of NfL and GFAP in disease progression, we measured their levels longitudinally. For patients treated with DMF, measurements were taken at baseline (T0) and 1 year ± 6 months (T1) from the start of treatment. For patients treated with OCRE, measurements were taken at baseline (T0), 1 year ± 6 months (T1), and 2 years ± 6 months (T2) from the start of therapy.

**Figure 6:**
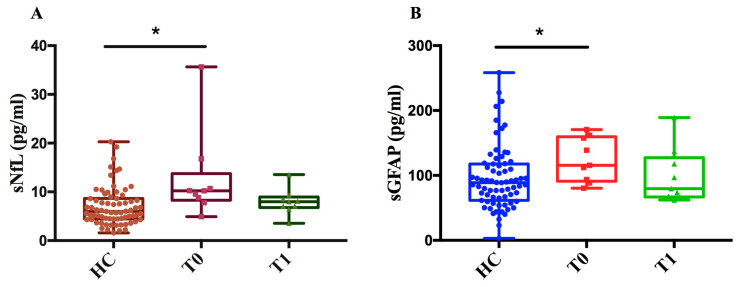
Measurement of sNfL and sGFAP levels in MS patients before and after treatment with DMF and controls. (**A**) sNfL (pg/mL) values of healthy controls (HCs) and patients at baseline (T0) and after one year ± 6 months of treatment (T1). (**B**) sGFAP (pg/mL) values of HCs and patients at time T0 and after one year ± 6 months of treatment (T1). The figure shows that, for both biomarkers, levels were significantly higher in patients at baseline (T0) compared to controls (sNfL * *p* = 0.01; sGFAP * *p* = 0.039) with a decreasing trend following treatment.

As of DMF, we measured sNfL and sGFAP levels in 9 MS patients at two time points (T0, T1) and in 71 healthy controls (HCs). We found that, at baseline (T0), sNfL and sGFAP levels in MS patients were significantly higher compared to those in healthy controls (*p* < 0.05) (Figure 6A,B). This difference between MS patients and healthy controls diminished after one year of treatment (T1), showing a decreasing trend in both NfL and GFAP levels after treatment compared to baseline.

Furthermore, as of OCRE, we measured sNfL and sGFAP levels in 20 MS patients at baseline (T0) and after about one (T1) and two (T2) years, comparing these results to those from 71 healthy controls (HCs). The results showed that, at baseline, before starting treatment, the patients’ group had significantly higher sNfL levels compared to the control subjects.

**Figure 7:**
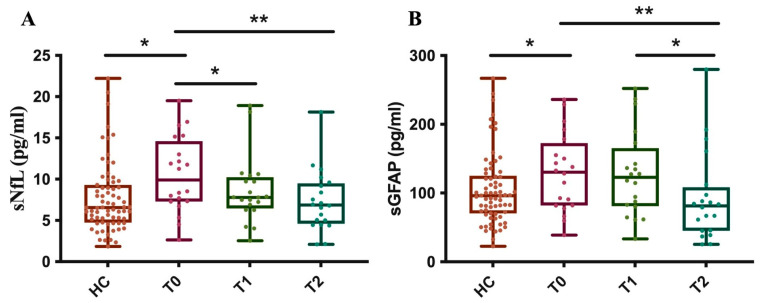
Longitudinal measurement of sNfL and sGFAP levels in OCRE-treated patients. The graph illustrates the levels of sNfL (**A**) and sGFAP (**B**) in MS patients at three specific time points, T0: baseline, T1: 1 year ± 6 months, and T2: 2 years ± 6 months after treatment with OCRE and compared to a healthy control group. (**A**) HC vs. T0 * *p* = 0.03; T0 vs. T1 * *p* = 0.03; T0 vs. T2 ** *p* = 0.007. (**B**) HC vs. T0 * *p* = 0.05; T0 vs. T2 ** *p* = 0.004; T1 vs. T2 * *p* = 0.012).

After just about 1 year of treatment (T1), a significant reduction in sNfL levels was observed in the patients. At this point, their average levels were no longer statistically different from those of the control group. The reduction in sNfL levels persists at time point T2, with the levels remaining stable and like those of the control group (Figure 7A).

These data indicate that Ocrelizumab treatment is effective in reducing sNfL levels in patients, bringing them into a range similar to that of healthy subjects after just one year and maintaining this effect over the long term.

Moreover, at baseline (T0), MS patients had significantly higher sGFAP levels compared to the control group. Unlike with sNfL, there is no statistically significant reduction in sGFAP levels at time point T1. Ocrelizumab treatment shows a significant reduction in sGFAP levels only after about 2 years of therapy (T2). At this point, the patients’ average levels drop to values similar to those of the control group (Figure 7B). These data suggest that the effectiveness of Ocrelizumab treatment on reducing sGFAP levels has a delayed effect, becoming significant only in the second year of therapy. This distinguishes the drug’s effect on axonal damage (NfL) from its effect on astroglial activation (GFAP).

## 3. Discussion

NfL and GFAP are the most promising biomarkers currently under investigation in multiple sclerosis (MS).

Despite their potential, significant challenges prevent routine use of NfL and GFAP in clinical practice. The main obstacle is the absence of generally accepted cut-off values, primarily because of numerous confounding factors that can potentially influence biomarker levels. More specifically, age and body mass index (BMI) are the most widely documented factors in the literature to date [15,16].

The age has been associated with increased levels of both NfL and GFAP in some studies [15,16,21].

Here, we describe our approach for determining cut-off values that can differentiate normal and pathologic sNfL and GFAP levels in patients, based on the data from 71 healthy controls (HCs) stratified by age.

Our results are comparable to those reported in the scientific literature, which demonstrates that the increase in sNfL levels becomes relevant in healthy subjects > 50 years old, and this elevation becomes statistically significant in the age group > 60 years [15,16,17].

Similarly to NfL, GFAP also showed a significant correlation with age (Pearson correlation r = 0.5258, R^2^ = 0.28, *p* < 0.0001). GFAP levels remained relatively stable up to the sixth decade (50–59 years) and increased significantly in healthy subjects > 59 years old.

Based on these data, we derived specific cut-off values consistent with the existing literature [17,22]. These cut-offs are platform- and cohort-specific and are intended for internal stratification only. External validation in larger, independent cohorts will be required before any clinical application. Our data indicate that the elevated sNfL and sGFAP levels observed in individuals above the age of 60 reflect an accelerated rate of underlying neuronal damage and astrogliosis as part of the ageing process. Consequently, age must be considered to establish reference values to discriminate between MS patients and healthy controls. Indeed, our data for these biomarkers were all adjusted for age, while BMI did not affect sNfL and sGFAP levels in our patient cohort, as also found in other studies in the literature [16] (Supplementary Data, Figure S1). However, the absence of correlation does not mean the absence of an effect, given the low sample size of the cohort of MS patients. Moreover, as all patients had preserved renal function, the potential impact of impaired renal clearance on serum sNfL and sGFAP levels is unlikely to be relevant in the present cohort.

In this study, we measured sNfL and sGFAP levels in 177 patients and 71 controls. We found significantly higher levels of both sNfL and sGFAP in MS patients compared to controls. Consistent with the literature data, the two biomarkers confirm their role as good markers of neuronal damage [23].

When dividing patients based on response or non-response to current treatment, both sNLF and sGFAP levels were higher in patients who did not respond to therapy, with statistical significance only for sNfL. To assess a difference in response related to the type of high- or low-efficacy treatment, we analyzed NR patients to LETAs and NR patients to HETAs separately. The analysis showed significantly higher levels of both sNfL and sGFAP in NR patients to LETAs. In patients treated with HETAs, only NfL levels were higher in NR patients compared to R patients, although this difference did not reach statistical significance. In our patient population, sGFAP was not a good indicator of non-response to HETAs therapy, likely because the levels of this biomarker were high in both R and NR patients due to the greater severity of the disease, also resulting from higher EDSS and MSSS values in patients treated with HETAs compared to LETAs. The data confirm that sGFAP levels increase with disease severity, and this association could mask a difference between responders and non-responders to the HETAs [10,24,25,26].

Because the many drugs used to treat multiple sclerosis have very different mechanisms, we analyzed a group of patients treated only with Dimethyl fumarate (a LETA drug) and a second group treated only with Ocrelizumab (a HETA drug) to assess whether they were able to predict a suboptimal response to treatment.

In our cohort, only sGFAP levels are higher in non-responders to DMF treatment than in responders, providing preliminary evidence that sGFAP may be associated with treatment non-response in DMF-treated patients. The difference in sGFAP levels between responders and non-responders remained significant even after adjusting for MSSS, suggesting that sGFAP can be considered as a dynamic treatment response biomarker. The absence of an increase in sNfL levels in DMF non-responders could be explained by the distinct temporal dynamics of the two biomarkers due to the fact that we sampled MS patients at least three months before or after a clinical relapse. Serum NfL is a well-established marker of acute or subacute neuroaxonal injury and it is known to rise transiently in association with clinical relapses and new inflammatory MRI activity, followed by a relatively rapid decline once acute inflammation resolves [19,27]. Because samples were not collected during the peak of inflammatory activity, transient increases in sNfL might not have been captured. In contrast, sGFAP, a marker of astrocytic activation and astrogliosis, has been shown to associate more strongly with chronic or progressive disease features [9] and may remain elevated beyond the period of acute inflammation. This differential temporal behaviour supports the complementary interpretation of sNfL and sGFAP as biomarkers of distinct pathological processes in multiple sclerosis. The selective increase in sGFAP in non-responders to DMF suggests that sGFAP could serve as a valuable biomarker to monitor treatment response, detecting persistent astrogliosis and chronic progression that DMF fails to control in these subjects.

Ocrelizumab significantly reduces relapsing disease activity and limits progression of disability in MS [14,28]; however, we did not find an increase in sNfL and sGFAP levels in patients classified as non-responders to Ocrelizumab. The lack of significant elevation in both sNfL and sGFAP in OCRE non-responders underscores the drug’s potent effect in suppressing inflammatory activity even in patients exhibiting clinical progression. This aligns with the literature suggesting that sNfL and sGFAP levels do not rise substantially in the absence of acute inflammation (Relapse-Associated Worsening, RAW) compared to patients reaching NEDA3 [29]. The non-response to Ocrelizumab mainly represents progression independent of relapse activity (PIRA), a neurodegenerative process that remains largely uncaptured by these established serum biomarkers despite the progression of disability, highlighting the need for novel biomarkers to detect ongoing CNS pathology under highly effective therapy [30,31].

Data in the current literature demonstrate that a reduction in NfL is observed with several MS disease-modifying therapies (DMTs) [6,7,27,32]. The effect of DMTs on sGFAP is not as uniform or marked as observed on sNfL [23,33].

Demonstrating the high efficacy of DMF and OCRE in suppressing disease activity, our patient cohort showed significantly lower levels of both sGFAP and sNfL after 1 year of treatment for DMF, and after 1 and 2 years of treatment for Ocrelizumab, compared to their respective baselines.

Specifically, we evaluated the levels of the two biomarkers after 1 year of treatment with Dimethyl fumarate and compared these levels with control subjects. We observed that both sNfL and sGFAP decreased after one year of treatment compared to baseline, reaching levels similar to the control group, as described in other cohorts [34].

For Ocrelizumab-treated patients, sNfL levels significantly decreased after 1 year and this reduction persisted through 2 years of treatment, with the levels stabilizing and becoming comparable to those of the control group, a finding consistent with the scarce data already present in the literature [35]. These data strongly indicate Ocrelizumab’s long-lasting efficacy in rapidly mitigating acute axonal damage, bringing sNfL levels into the healthy range within the first year.

Reduction in sGFAP was evident after 2 years of treatment with OCRE, suggesting a more delayed mechanism of action on astrogliosis. Based on our data, we cannot rule out whether this effect of delayed decrease in GFAP levels by Ocrelizumab is due to the “turnover” of a stable glial scar or the resolution of persistent astrocyte reactivity.

Finally, based on the established healthy cut-off values, we observed that in our total patient cohort only 27% exceeded the control cut-off values for sNfL and 13% for sGFAP. This result could be attributed to the stable phase of the disease in the patients analyzed and the high proportion receiving effective DMTs.

Based on these data, the necessity remains to establish more appropriate cut-off values for use in diagnosis.

One limitation of our study is related to the sample size and cohort composition. While the overall cohort is substantial, the size of the subgroups analyzed (DMF R vs. NR; OCRE R vs. NR) may be limited, potentially reducing the statistical power to detect smaller differences. These findings should be interpreted as exploratory and hypothesis-generating and require confirmation in larger, independent cohorts. Moreover, grouping all low-efficacy therapies (LETAs) together might introduce heterogeneity, as these drugs do not share identical mechanisms of action, which could influence the biomarker results. A Supplementary Table (Table S1) reported sNfL and sGFAP levels stratified by individual drug class. Furthermore, based on the cross-sectional nature of the study, we cannot rule out the occurrence of reverse causation in the interpretation of our findings.

In conclusion, sGFAP may represent a valuable biomarker to monitor response to DMF therapy, likely by detecting persistent astrogliosis.

Ocrelizumab shows a rapid effect on axonal damage (sNfL) but a delayed effect on astroglial activation (sGFAP), highlighting a distinction in the drug’s action on these two pathological processes. While sGFAP shows promise in early disease, its correlation with advanced disease severity means that high baseline levels in responders may mask the therapeutic effect, leading to similar values between R and non-R patients.

## 4. Materials and Methods

### 4.1. Study Design and Participants

This study was a retrospective, observational study approved by the local Ethics Committee of ASST Santi Paolo e Carlo, San Paolo Hospital of Milan. All patients and controls provided written informed consent before their inclusion. The study involved 177 patients and 71 healthy controls recruited between July 2022 and February 2025.

Inclusion criteria were a diagnosis of MS according to revised McDonald criteria [36], age between 18 and 70 years, a minimum follow-up period of 6 months, and receiving treatment for a minimum of two years. The patients were free of clinical relapses during the time of baseline sampling.

Patients were classified as responders or non-responders to current therapy based on “No Evidence of Disease Activity 3” (NEDA-3) status during two years of treatment. NEDA-3 was defined as the absence of relapses, absence of new or enlarging T2 lesions or gadolinium-enhancing lesions on MRI, and absence of confirmed disability progression. We classified MS patients as either Rs or NRs based on NEDA-3 assessment during the 2-year follow-up period at our centre. The follow-up on treatment was calculated as 1 year before the first visit and 1 year after the first visit. If a patient was in clinical relapse during the first visit at our centre, we postponed the inclusion in the study by the next neurological visit (3 months after) in order to have patients in a stable clinical disease. If a patient started a treatment less than 1 year before our first visit at our centre, we calculated the 2-year period, prolonging the follow-up period after the visit.

Moreover, the administered DMTs were classified into low/moderate-efficacy treatment agents (LETAs): Interferons (*n* = 8), Glatiramer acetate (*n* = 8), Teriflunomide (*n* = 17), Dimethyl fumarate (*n* = 32), Azathioprine (*n* = 2), Methotrexate (*n* = 1), and high-efficacy treatment agents (HETAs): modulators of the sphingosine-1-phosphate receptor (Fingolimod (*n* = 15), Siponimod (*n* = 3)), Cladribine (*n* = 3) and monoclonal antibodies targeting CD20 (Ocrelizumab (*n* = 51), Rituximab (*n* = 4)), CD52 (Alemtuzumab (*n* = 1), and α4-integrin (Natalizumab (*n* = 7)). This classification was adopted as a pragmatic, clinically oriented grouping designed to reflect treatment escalation strategies rather than mechanistic similarities.

We then selected two patient subgroups. The first group consisted of 24 patients treated with Dimethyl fumarate (LETAs); 9 of these patients were sampled at baseline before the treatment (time point T0) and 1 year ± 6 months after treatment (time point T1). The second group consisted of 49 patients treated with Ocrelizumab (HETAs); 20 of these patients were sampled at baseline before the treatment (time point T0), 1 year ± 6 months (time point T1) and 2 years ± 6 months (time point T2) after treatment.

### 4.2. Sample Collection and Serum sNfL and sGFAP Quantification

Blood samples were collected before the start of Ocrelizumab and Dimethyl fumarate treatment, and again after one and two years. For all patients on other treatments, only a single blood sample was taken during treatment.

Peripheral blood samples were obtained from healthy controls (HCs) and MS patients, by means of serum-collection tubes. Samples were spun at 2000× *g* for 10 min. Subsequent to processing, the serum was harvested, divided into 400 µL aliquots, and maintained at −80 °C until the time of assay. Serum concentrations of NfL and GFAP were measured via the Neurology 2-Plex B Kit (Quanterix, Billerica, MA, USA) on the SIMOA™ SR-X platform, an ultrasensitive immunoassay. All procedures strictly adhered to the manufacturer’s analytical protocols. The assay performance parameters for NfL included a lower limit of quantification (LLOQ) of 0.400 pg/mL and a limit of detection (LOD) of 0.071 pg/mL. For the GFAP assay, the LLOQ and LOD were established at 4.15 pg/mL and 0.410 pg/mL, respectively.

### 4.3. Statistical Analyses

We performed statistical analyses with GraphPad Prism 10.3.1 software, SPSS V. 29.0.1.0, and R 4.4.1 packages limma and sva 3.54.0 [PMID:22257669]. Spearman test was used to assess the correlation between NfL and GFAP in relation to putative confounding factors such as age, batch, and BMI. One-way ANOVA and Bonferroni’s multiple comparisons test were used to calculate the association between the two biomarkers and age stratified by decades. Biomarker values were log_10_-transformed to approximate normality (Shapiro–Wilk test) and cut-off values were derived as mean + 2 SD of the transformed data. Multiple linear regression was used to calculate differences in NfL and GFAP levels across the various study groups, using age and batch as covariates. We performed ANCOVA with age as a covariate to calculate the difference in NfL and GFAP levels in patients at baseline and after one or two years of treatment. The MSSS was calculated using the R package MS.sev. Linear regression analyses were performed to investigate the association between sNfL and sGFAP levels and clinical disability and disease severity, adjusting for age. logFC represents the change in log2-transformed protein levels per unit increase in disability/severity index; AveExpr denotes the mean log2-expression across the cohort. *p*.Value and adj.*p*.Val indicate nominal and Benjamini–Hochberg-adjusted significance levels, respectively. Prior batch effect correction was performed using the ComBat method implemented in sva library, eliminating the unwanted systematic variation [PMID:16632515]. *p* ≤ 0.05 was considered statistically significant.

## Figures and Tables

**Table 1:** Cut-off values for sNfL levels stratified by age decades. For each decade, it provides the mean, standard deviation (SD), median, range, and cut-off values in pg/mL, which were calculated as the mean + 2 SD. Cut-off values were calculated on log_10_-transformed data and subsequently back-transformed to the original scale for clinical interpretation.

Age (Years)	N	Mean sNfL (pg/mL)	SD (pg/mL)	Median sNfL (pg/mL)	Range (pg/mL)	Mean log_10_ sNfL (pg/mL)	SD of log_10_ sNfL	log_10_ Cut-Off (Mean + 2SD)	Final Cut-Off (pg/mL)
20–29	7	5.1	1.3	5.07	3.93–7.65	0.7	0.1	0.9	7.8
30–39	21	5.0	1.6	4.97	2.29–7.74	0.7	0.2	1.0	9.4
40–49	14	6.4	1.8	6.39	3.29–9.17	0.8	0.1	1.1	11.5
50–59	14	8.6	2.2	9.08	4.67–11.08	0.9	0.1	1.2	14.7
≥60	15	12.8	4.1	11.34	7.01–21.31	1.1	0.1	1.3	19.4

**Table 2:** Cut-off values for sGFAP levels stratified by age decades. For each decade, it provides the mean, standard deviation (SD), median, range, and cut-off values in pg/mL, which were calculated as the mean + 2 SD. Cut-off values were calculated on log_10_-transformed data and subsequently back-transformed to the original scale for clinical interpretation.

Age (Years)	N	Mean sGFAP (pg/mL)	SD (pg/mL)	Median sGFAP (pg/mL)	Range (pg/mL)	Mean log_10_ sGFAP (pg/mL)	SD of log_10_ sGFAP	log_10_ Cut-Off (Mean + 2SD)	Final Cut-Off (pg/mL)
20–29	7	77.7	16.9	76.82	55.8–109.0	1.9	0.1	2.1	116.3
30–39	21	89.1	34.8	86.81	31.1–156.3	1.9	0.2	2.3	189.1
40–49	14	85.9	26.8	93.49	35.4–129.7	1.9	0.2	2.2	174.9
50–59	14	117.3	42.4	106.32	67.8–205.9	2.0	0.2	2.3	221.6
≥60	15	147.5	65.3	132.83	82.2–294.2	2.1	0.2	2.5	301.0

**Table 3:** Patient and healthy control demographic and clinical data.

Variables	MS Patients	HCs	*p*-Value
Numbers	177	71	
Treated (%)	152 (86%)		
R/NR	114 (75%)/38 (25%)		
-LETAs	68 (45%)		
R/NR	48 (71%)/20 (29%)		
-HETAs	84 (55%)		
R/NR	66 (79%)/18 (21%)		
Untreated (%)	25 (14%)		
Female (%)	112 (63%)	49(69%)	
Male (%)	65 (37%)	22 (31%)	
Age at sampling, mean ± SD	45 ± 13	46.25 ± 13	ns
Disease duration, mean ± SD	12 ± 9.7		
Type of MS:			
RRMS	167 (94.35%)		
PPMS	4 (2.25%)		
SPMS	6 (3.4%)		
EDSS median (min–max)	2.5(0–8.5)		
MSSS median (min–max)	3.86 (0.14–9.7)		
NfL levels, mean (pg/mL) ± SD	9.6 ± 6.5	8.10 ± 4.65	*p* = 0.024
GFAP levels (mean (pg/mL) ± SD	125 ± 78.2	99.6 ± 49.5	*p* = 0.004

ns: not significant.

**Table 4:** Linear regression analysis of NfL and GFAP levels in relation to disease severity. Linear regression shows that, as disability (EDSS) and disease severity (MSSS) increase, GFAP levels tend to rise in a non-statistically significant manner.

**MSSS**				
	logFC	AveExpr	*p*.Value	adj.*p*.Val
GFAP	2.798309	122.5094	0.199568	0.199568
NfL	0.3001	9.886128	0.097441	0.194881
**EDSS**				
	logFC	AveExpr	*p*.Value	adj.*p*.Val
GFAP	4.971869	122.5094	0.093438	0.123436
NfL	0.379818	9.886128	0.123436	0.123436

**Table 5:** Differences between groups of responders and non-responders in baseline characteristics.

Variables	Responder (R)	Non-Responder (NR)	*p*-Value
Numbers	114 (75%)	38 (25%)	
Female/Male	72/42	22/16	
*LETAs*	48 (42%)	20 (52.6%)	
*HETAs*	66 (58%)	18 (47.4%)	
Age at sampling, mean ± SD	44.9 ± 11.3	46.1 ± 12.9	ns
Disease duration, mean ± SD	12.2 ± 9.4	11.2 ± 9.9	ns
Type of MS:			
RRMS	107	36	
PPMS	2	2	
SPMS	5	0	
EDSS median (min–max)	2.5 (0–7.5)	3.5 (1–8.5)	*p* < 0.001
NfL levels, mean (pg/mL) ± SD	8.4 ± 4.1	10.6 ± 5.4	*p* = 0.007
GFAP levels (mean (pg/mL) ± SD	119 ± 85.4	134.6 ± 73.9	ns

ns: not significant.

**Table 6:** Characteristics of patients treated with LETAs or HETAs.

Variables	LETAs R	LETAs NR	*p*-Value	HETAs R	HETAs NR	*p*-Value	*p*-Value LETAs vs. HETAs
Numbers	48 (70.6%)	20 (29.4%)		66 (78.6%)	18 (21.4%)		
Female (%) Male (%)	69% 31%	55% 45%	ns	59% 41%	61% 39%	ns	ns
Age at sampling, mean ± SD	45 ± 11	48 ± 14	ns	45 ± 11	44 ± 11	ns	ns
Disease duration, mean ± SD	11.5 ± 9	15 ± 11	ns	13 ± 9	9 ± 7	ns	ns
NfL mean (pg/mL) ± SD	8.4 ± 4.3	11.6 ± 5.8	*p* = 0.024	8.32 ± 4	9.6 ± 5	ns	
GFAP mean (pg/mL) ± SD	99.7 ± 55	143 ± 62.7	*p* = 0.009	132.7 ± 100	125 ± 85.5	ns	
EDSS median (min–max)	2 (0–8.5)			3 (1–7.5)			*p* < 0.001
MSSS median (min–max)	3 (0.14–9.77)			5.24 (0.72–9.57)			*p* = 0.001

ns: not significant.

**Table 7:** Characteristics of patients treated with DMF or OCRE. DMF: Dimethyl fumarate; OCRE: Ocrelizumab; R: responder; NR: non-responder.

Variables	DMF R	DMF NR	*p*-Value	OCRE R	OCRE NR	*p*-Value	*p*-Value DMF vs. OCRE
Numbers	18 (77%)	6 (23%)		38 (81%)	11 (19%)		
Disease duration, mean ± SD	7.4 ± 8.6	5.3 ± 4.7	ns	11.75 ± 9.5	8.8 ± 8	ns	*p* < 0.05
Age at sampling, mean ± SD	39 ± 11.5	35 ± 6.5	ns	44 ± 10	44 ± 10	ns	*p* < 0.05
NfL mean (pg/mL) ± SD	6.53 ± 2.68	6.14 ± 2.4	ns	7.1 ± 3.8	7.8 ± 3.7	ns	
GFAP mean (pg/mL) ± SD	89.3 ± 40.7	132.5 ± 47.8	*p* = 0.036	118 ± 65.6	117 ± 75.8	ns	
EDSS median (min–max)	1.5 (0–2.5)	2.5 (1–6)	ns	3.75 (1–7.5)	3 (1.5–6.5)	ns	*p* < 0.0001
MSSS median (min–max)	2.6 (0.43–5.45)	4.7 (1.93–9.4)	*p* < 0.01	5.45 (1.4–9.4)	6.6 (1.1–9.6)	ns	*p* = 0.0001

ns: not significant.

## Data Availability

The raw data can be obtained from the corresponding author upon request.

## Supplementary Materials

The following supporting information can be downloaded at: https://www.mdpi.com/article/10.3390/ijms27031441/s1.
