# Supplementary material for: Serum Neurofilament Light Chain and Glial Fibrillary Acidic Protein as Differential Biomarkers of Response to Dimethyl Fumarate and Ocrelizumab in Multiple Sclerosis
Source: Int J Mol Sci. 2026 Jan 31;27(3):1441. doi: 10.3390/ijms27031441 (PMC12897660; doi:10.3390/ijms27031441)
Supplement: Supplementary file 1 [file ijms-27-01441-s001.zip › Supplemetary data-rev.pdf]

Supplemetary data

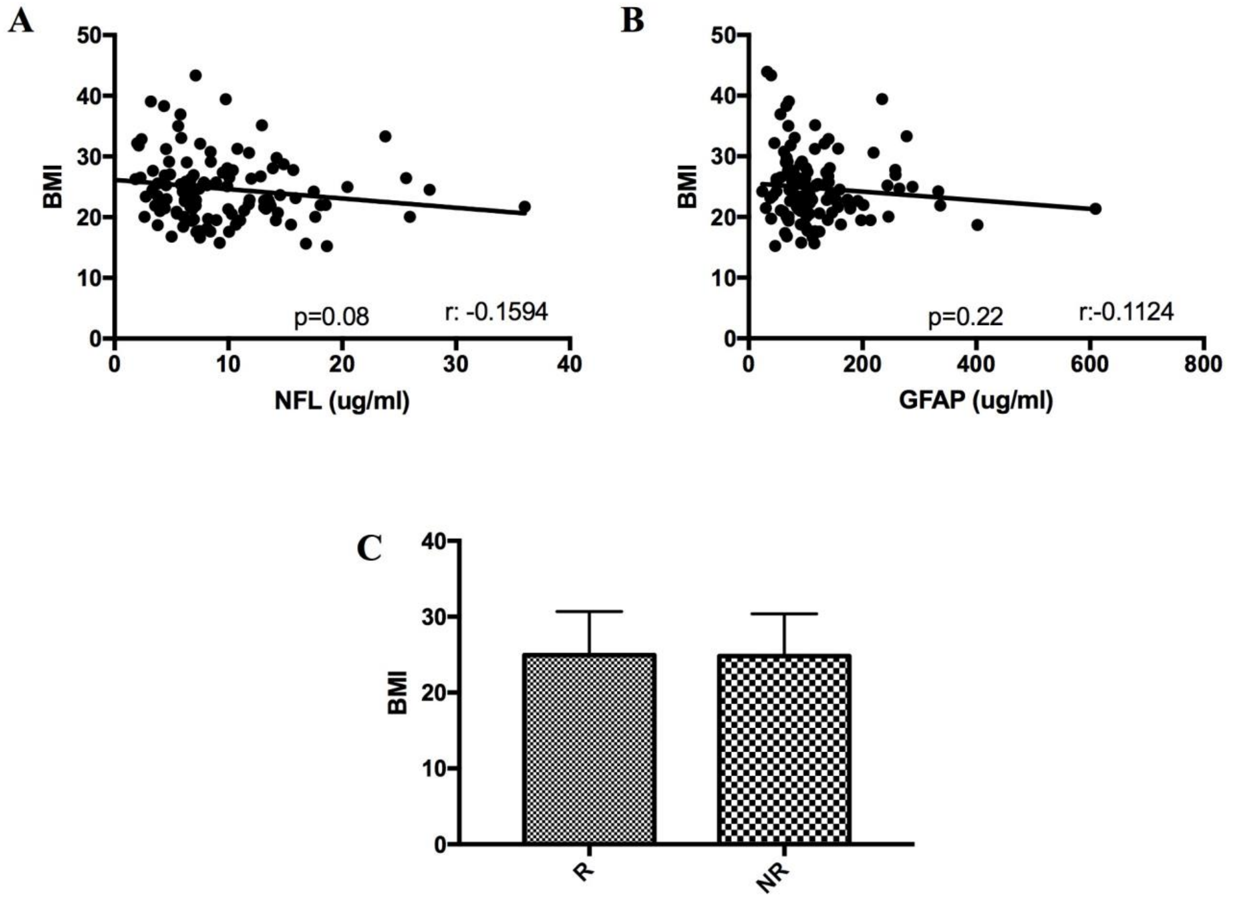

Figure S1. sNfL and sGFAP association with BMI. Correlation between NfL (**A**) and GFAP (**B**) levels with BMI in 119 MS patients. A significant inverse correlation was not detected within our patient cohort (Pearson correlation r = -0.1594, R2=0.026 p < 0.08 for NfL) (Pearson correlation r = -0.1124, R2=0.0106, p < 0.22 for GFAP). Panel **C** showed the difference in BMI between patients responder and non-responder to treatment. No difference was found between the two groups.

Table S1. Serum sNfL and sGFAP levels across individual disease-modifying therapies and untreated patients. Values are shown as mean ± standard deviation. These data are provided for descriptive purposes only.

Treatment	*sNfL (pg/ml)*	*sGFAP (pg/ml)*
Azatioprina (n=2)	31,51 ±5,42	165,31 ±7,63
Dimetilfumarato (n=32)	7,82 ±3,85	102,93± 44,69
Glatiramer Acetato (n=8)	9,41 ± 5,79	133,71 ±102,99
Interferone (n=8)	10,69 ± 7,78	106,62 ±35,19
Metotrexato (n=1)	12,62	233,07
Teriflunomide (n=17)	11,19 ± 7,23	116,07 ±41,25
Cladribina (n=3)	8,25 ± 1,59	82,26 ±23,9
Fingolimod (n=15)	7,37 ± 4,09	108,55 ±70,12
Alemtuzumab (n=1)	18,41±	86,88
Natalizumab (n=7)	9,44 ± 7,33	247,82 ±195,52
Ocrelizumab (n=51)	9,12± 6,03	133,80 ±81,55
Rituximab (n=4)	6,02± 3,89	109,86 ±49,70
Siponimod (n=3)	11,18± 1,72	104,26 ±46,15
Not treated (n=25)	11,52 ± 8,20	124,63 ±65,55
